# Mutagenic Distinction between the Receptor-Binding and Fusion Subunits of the SARS-CoV-2 Spike Glycoprotein and Its Upshot

**DOI:** 10.3390/vaccines9121509

**Published:** 2021-12-20

**Authors:** Robert Clark Penner

**Affiliations:** 1 Institut des Hautes Etudes Scientifiques, 35 Route des Chartres, 91440 Bures-sur-Yvette, France; rpenner@ihes.fr; 2 Mathematics Department, University of California at Los Angeles, Los Angeles, CA 90095, USA

**Keywords:** SARS-CoV-2 spike, mutagenic pressure, backbone free energy, vaccinology

## Abstract

We observe that a residue R of the spike glycoprotein of SARS-CoV-2 that has mutated in one or more of the current variants of concern or interest, or under monitoring, rarely participates in a backbone hydrogen bond if R lies in the S${}_{1}$ subunit and usually participates in one if R lies in the S${}_{2}$ subunit. A partial explanation for this based upon free energy is explored as a potentially general principle in the mutagenesis of viral glycoproteins. This observation could help target future vaccine cargos for the evolving coronavirus as well as more generally. A related study of the Delta and Omicron variants suggests that Delta was an energetically necessary intermediary in the evolution from Wuhan-Hu-1 to Omicron.

## 1. Introduction

This short note isolates a specific and elementary observation about Protein Data Bank (PDB) [1] files concerning the mutated residues in the current variants of concern and of interest, plus the variants under monitoring, as per [2] 22 October 2021, of the SARS-CoV-2 spike glycoprotein S. This observation is then applied to the genesis of the Omicron variant. It has not, to our knowledge, appeared in the literature other than in our own earlier work [3] in the context of specific variants of concern, and it may be material going forward in designing mRNA or other types of vaccine cargos, if necessary, as the coronavirus continues to evolve. It is worth consideration, in this now highly studied example, as a potentially more general example of viral glycoprotein mutagenesis since we provide a partial explanation for our observation based upon general principles involving free energy.

To state this fact about mutagenic pressure in the spike, recall [4] that, as in many examples of viral glycoproteins, in particular commonly with Class I fusion mechanisms [5,6], S is composed of the two subunits S${}_{1}$ and S${}_{2}$, where S${}_{1}$ mediates receptor-binding extracellularly and S${}_{2}$ mediates fusion within an endosome. One particularity of S is a host-furin-mediated cleavage between S${}_{1}$ and S${}_{2}$ at residue number 685–686. There is, furthermore, a second cleavage lying adjacent to the fusion peptide, mediated by the host-cathepsin or serine protease of a C-terminal segment $S_{2}^{\prime }$ of S${}_{2}$ at residue number 815–816. See [7] for more information on cleavage in the SARS-CoV-2 spike.

In any protein, hydrogen bonds form between backbone nitrogen atoms N${}_{i}$-H${}_{i}$ and oxygen atoms O${}_{j}$=C${}_{j}$ in different peptide units and these are called backbone hydrogen bonds (or BHBs). (To be precise, a DSSP [8] hydrogen bond is accepted as a BHB provided that the distance between H${}_{i}$ and O${}_{j}$ is less than 2.7 Å and both ∠NHO and ∠COH each exceed 90${}^{\circ }$). A protein residue R${}_{i}$ itself is said to *participate* in a BHB if either the nearby nitrogen N${}_{i}$-H${}_{i}$ donates or the nearby oxygen O${}_{i}$=C${}_{i}$ accepts a BHB. (Again, to be precise, if at least two monomers of the trimeric spike participate, then the residue itself participates.) On average for all proteins, roughly 70–80% of all residues participate in a BHB [9].

Here is the main easily confirmed empirical observation of this paper, which is quantified in Table 1 and subsequently discussed: A residue R of the SARS-CoV-2 spike glycoprotein S, which has mutated in one or more of the current variants of concern or interest, or under monitoring [2] (cf. Table 1 for these mutagenic residue numbers), rarely participates in a BHB if R lies in $S_{1}$ and usually participates in a BHB if R lies in $S_{2}$.

A general but not entirely satisfactory explanation for this involves the free energy of structural details stabilized by BHBs. In particular, viral glycoproteins, which mediate receptor-binding and membrane fusion, are by their very nature metastable. It follows that successful viral mutation can neither increase free energy by so much as to disturb the stability of the molecule nor decrease it by so much as to interrupt near-instability, for otherwise the molecule will, respectively, either explode or fail to reconform and function correctly. The minimal way to avoid this twofold constraint is to mutate residues that do not participate in BHBs at all, and that is precisely what we find in S${}_{1}$ before mutation. However, we shall also discover, most interestingly, that this is not reversible.

We shall discuss S${}_{2}$ subsequently only after including certain salient definitions, facts, and data, and note in this introduction just that the existence of BHBs and their free energies are obviously functions of pH. This alone might account for differences between $S_{1}$ and S${}_{2}$ since the endocytotic pathway is highly acidifying [4].

## 2. Materials and Methods

As is customary, we recorded mutations relative to an original Wuhan genome called Wuhan-Hu-1 and its corresponding spike protein (UniProt [10] Code P0DTC2) by considering only structure files with resolutions below some bound; for our purposes, this included a resolution of at most 3.0 Å, neither cleaved nor bound to an antibody or receptor, and computed via cryo-electron microscopy. These 15 exemplar structures 6VXX 6X29 6X79 6XLU 6XM0 6XM3 6XM4 6ZB5 6ZGE 7A4N 7AD1 7DDD 7DF3 7DWY and 7JWY for S from the PDB depend upon various techniques of stabilizing S in its prefusion conformation [11,12,13,14,15,16,17,18,19,20]. The molecules are therefore not truly identical, hence the utility of taking consensus and average data across the collection of PDB files, as we shall do.

Some of the previous considerations can be calibrated by employing a new concept and quantity in structural biology, namely the so-called backbone free energy (BFE) from [21], which can be computed from a PDB file to be called simply a *structure*. Roughly, the BFE of a structure stabilized by a BHB is computed from geometry [22] by comparing the planes containing the peptide units of the donor and accepter of the BHB, and by applying the Pohl–Finkelstein quasi Boltzmann Ansatz [23,24,25].

Let us next briefly give a more complete discussion of the method from first principles, referring the interested reader to [22] for the background and data for general proteins (as explained here), ref. [21] for application to viral glycoproteins, ref. [26] for application to coronavirus spikes, and [3] for the SARS-CoV-2 spike S in particular. One starts by choosing a suitably unbiased subset of the PDB and computing all of its attendant BHBs, comprising a collection of 1,166,165 BHBs for the unbiased subset in [22] and for our discussion throughout. For each of these BHBs, there is a rotation of space from the peptide plane of its donor to the peptide plane of its receptor, mapping the peptide bond of the former to that of the latter. This defines an a priori distribution on the space of all rotations, which is computed once and for all. Now given another BHB, there is an associated free energy given by taking the log density of this a priori distribution at this new subject BHB, which is suitably normalized to approximate the BFE in kcal/mole. Thus, the geometry of the backbone described in a PDB file determines a BFE associated to each BHB in any protein. The fundamental fact, established in [21] for viral glycoproteins, is that residues of large BFE target locations of large conformational change in the backbone, typically including, in particular, the fusion peptide.

There is a trichotomy of possibilities for a residue R in a specific structure: R may be modeled in the structure and participate in a BHB or not, and in this latter case, we say R is *absent*, but R may also simply be *missing* from the PDB file. (As before, these properties of residues are taken as consensus data from the three monomers.) R can be missing for a number of simple reasons: the protein may be disordered at R [4]; the experiment may be inaccurate or problematic at R; the data and its refinement may not model R within reasonable parameters; or R may be C-terminal or N-terminal to the experimentally synthesized sub-peptide of the protein S.

The average of the resolutions in our collection of structures is 2.77 Å and of the percentages of Ramachandran outliers is 0.1, thus these are all high-quality experimental structures. Note that clashscores and sidechain outliers are not particularly relevant measures of quality for our purposes. As argued in [3], it follows that the first among the possibilities for R missing is the most likely, thus one might *conflate missing with disordered* for high-quality structures within the PDB-range. The consensus range of our collection of structures for S is comprised of residue numbers 27 to 1147.

A residue that is missing or absent is said to be *unbonded* and is *bonded* otherwise. If a residue R${}_{i}$ is bonded, then it participates in a BHB, thus there is either a BHB with donor N${}_{i}$-H${}_{i}$ or one with acceptor O${}_{i}$=C${}_{i}$, or both, and the BFE of the residue R${}_{i}$ is defined to be the maximum of the BFEs of these one or two BHBs, first averaged over the two or three monomers. If a residue is unbonded, then its BFE is undefined.

Specifically to give a quantitative sense to what follows, the range of BFE values is −2.9 to +6.85 kcal/mole with approximate 50th, 90th, and 99th percentile cutoffs given by 1.4, 4.6, and 6.6, respectively. The validated hypothesis is that if the BFE of a residue lies in the 90th percentile, i.e., is at least 4.6 kcal/mole, then within one residue of it along the backbone, the sum of the two adjacent backbone conformational angles changes by at least 180 degrees in its pre to post-fusion reconformation. The converse does not hold.

## 3. Results

### 3.1. Wuhan-Hu-1 to Many Variants

**Figure 1:**
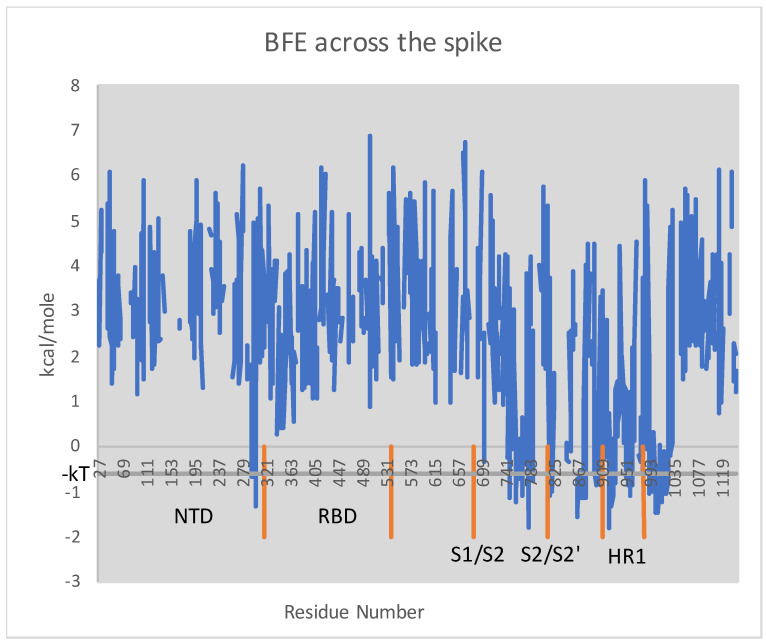
Plot of BFE by the residue across the PDB-covered spike, namely residues 27–1147 of S. Illustrated in orange are the respective residue ranges for the N-terminal domain, the receptor-binding domain, the $S_{1}/S_{2}$ cleavage, the $S_{2}/S_{2}^{\prime }$ cleavage, and the first heptad repeat domain. The gray horizontal line indicates one “heat quantum” kT≈0.6kcal/mole below zero. One can confirm by comparison with the structure itself that the intersections of BFE with this line corresponds to α helices and, in fact, ones whose backbone geometry is especially near ideal α helices according to considerations of free energy.

As argued before, in order to preserve the metastability of the molecule, the BFE before and after a mutation must be more or less constant across the spike. The plot of BFE across the spike is depicted in Figure 1. Higher BFE is evidently concentrated in $S_{1}$ compared to $S_{2}$. The several regions of meaningful negative BFE are illustrated in the figure by the intersections of the plot with the gray horizontal line, which corresponds to nearly ideal α helices. Notice that each cleavage site is surrounded by a region of high BFE, and the same is true for the two ends of HR1.

**Figure 2:**
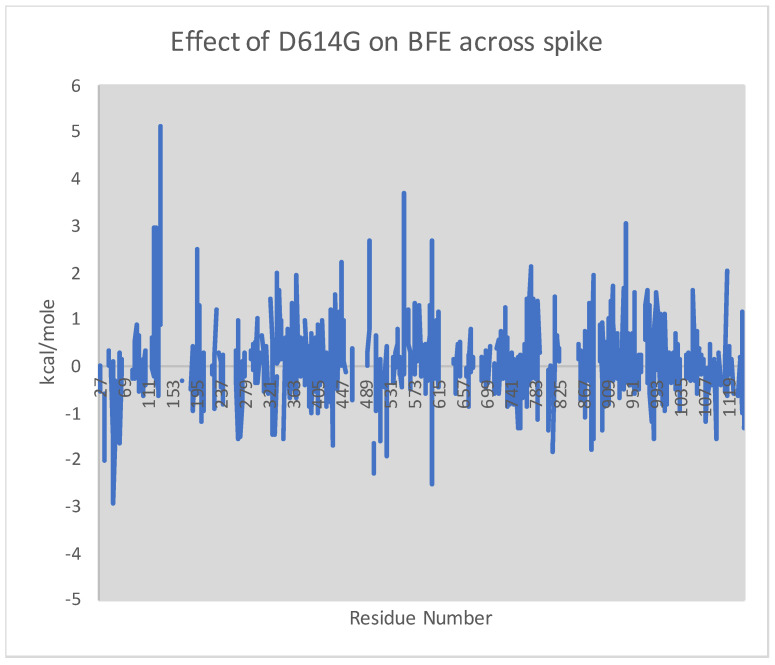
Comparison of BFE across the spike from the single mutation D614G [27]. The BFE of Wuhan-Hu-1 is computed at each residue as the average of PDB structures 7KDG and 7KDH, which are stabilized in the prefusion conformation by mutations R682G, R683G, and R685G, plus the 2P mutation given by K986P and V987P; the BFE of the D614G mutation is computed as the average of structures 7KDK and 7KDL, analogously stabilized but also with the D614G mutation. In each case, missing or absent residues give null. Plotted is the difference of the former minus the latter. The Wuhan-Hu-1 BFE at residue 614, itself, is 2.26 kcal/mole compared to 2.12 kcal/mole for D614G, but despite this near equality at residue 614, the BFE across the entire spike is altered.

However, as depicted in Figure 2, the single mutation D614G, which quickly globally overtook Wuhan-Hu-1 as the predominant strain, alters BFE along the entire backbone by as much as 5.10 kcal/mole at residue 134, whereas by only 0.14 kcal/mole at residue 614. Thus, a single local change of the primary structure can engender a long-range change of BFE across the whole spike glycoprotein.

Table 1 presents findings and data about the mutating residue numbers M common to one or more of the variants under consideration here. Specifically, the table summarizes BFEs and numbers of absent, missing, and unbonded residues in each of the molecules $S,S_{1}$, and $S_{2}$, as well as in their respective intersections $\bar{S}=S\cap M,{\bar{S}}_{1}=S_{1}\cap M$, and ${\bar{S}}_{2}=S_{2}\cap M$, with the mutagenic residue M under consideration.

Several trends present themselves:

- $S_{1}$ is more disorganized than $S_{2}$ (i.e., # missing is larger);
- there are more loops in $S_{1}$ than $S_{2}$ (i.e., # absent is larger);
- the BFE of $S_{1}$ is larger than $S_{2}$;
- the same three assertions above hold for ${\bar{S}}_{1}$ compared to ${\bar{S}}_{2}$; and
- a greater ratio $\frac{43}{655}\approx 0.066$ of residues in $S_{1}$ are mutating in the variants under consideration than $\frac{13}{460}\approx 0.028$ in $S_{2}$.

Moreover, this table quantifies our main new

**Basic Finding**: The residues mutating in the variants under consideration are more often unbonded in $S_{1}$ and bonded in $S_{2}$.

Note that the missing and absent columns in the table come directly from the PDB and DSSP, with no provisos (other than those conventions in parentheses in the text). The unbonded column presents our novel insights and depends upon a cutoff 5; in particular, it is bonded, i.e., neither absent nor missing, in at least five of the 15 structures. The last BFE column depends not only on the cutoff 5 but also on our theory of BHBs. All of the preceding trends are invariant under changing this cutoff by unity, with this data not presented.

### 3.2. Wuhan-Hu-1 to Delta to Omicron

We shall quantify the basic finding for the three mutational steps from Wuhan-Hu-1 (W) to Delta (Δ), W to Omicron (*O*), and Δ to *O*. The geometry of the spike for Δ is derived from the PDB files 7V7O 7V7P…7V7V in the same manner as before for W from its exemplar structures and again with a cutoff of five.

Table 2 quantifies our basic finding under various scenarios, with the Wto(∗) and ΔtoO mutations comparable, where (*) is the union of the variants considered in the previous section. The WtoO transition is anomalous with its much smaller percentage of unbonded mutated residues. The explanation follows from the last column, showing the large percentages of residues that are bonded in W but not in Δ, and hence of higher mutagenic potential for their transition to *O* according to the basic finding.

The supposition that Δ played an intermediary role in the passage to *O* is already strongly bolstered by the nearly complete dominance of Δ in South Africa before the advent of *O*. Meanwhile, the PDB files 7LYK 7LYL…7LYQ [28] for the earlier South African β variant are of a lower quality, thus their interpretation is problematic and not fully presented here; however, the resulting ${\bar{S}}_{1}$ entry for β to *O* in Table 2 is 48%.

## 4. Discussion

We find that mutagenic pressure on $S_{1}$ exceeds that on $S_{2}$, as expected based on the function and location of both subunits, and that the former is more disorganized and with a lower percentage of bonded residues than the latter. These findings are consistent with the general trend that certain B-factors [29] in the receptor-binding subunit usually exceed those in the fusion subunit of a viral glycoprotein, at least in the prefusion conformation.

It is argued that the mutation of unbonded residues avoids the twofold constraint on BFE imposed by the metastability of the viral glycoprotein, thus explaining the tendency of mutating residues in $S_{1}$ to be unbonded. However, among the mutating residues (19) 156–158, 452, 478, 614, 681, and 950 defining Delta, only 614 and 950 are bonded in Wuhan-Hu-1, in line with the basic finding, while only 681 is **unbonded** in Delta. This is fascinating and shows that there is more to our energetic argument than simply mutations avoiding BHBs. On the contrary, Wuhan-Hu-1 to Delta is not reversible, and there is thus an evolutionary dynamics of fixing BHBs for function and erasing them to enhance mutation in light of the basic finding, at least in this case of Wuhan-Hu-1 to Delta. In contrast, among the mutated residues 80, 215, 417, 484, 501, 614, and 701 defining Beta, only 417, 614, and 701 are bonded in Wuhan-Hu-1, while these plus 484 are bonded in Beta, here using any cutoff greater than unity, so bonded mutagenic residues remain bonded in this case. Backbone hydrogen bonds therefore provide an additional level of regulation of viral mutation, and this warrants further study.

As was already mentioned, the different pH of activation for the two subunits $S_{1}$ and $S_{2}$ may explain the opposite trend in the latter that mutating residues tend to be bonded since the prefusion-stabilized spike structures may better reflect the actual geometry and consequent BHBs of $S_{1}$ compared to $S_{2}$. Another related possibility is that pre-cleavage $S_{1}$ sits on top of $S_{2}$ as a kind of cap, thereby sterically constraining the latter, thus the active geometry of $S_{2}$ is displayed only post-cleavage and in the course of acidic post-fusion reconformation.

In any case, the findings on $S_{1}$ suggest a strategy for anticipating residues primed for mutation therein. However, going forward, it is the residues that are unbonded for the currently mutated variants, rather than for Wuhan-Hu-1, that should be considered as likely future candidates, just as in our analysis of Delta to Omicron.

## 5. Conclusions

The new and potentially more general insight is that protein secondary structure may provide a regulatory network controlling mutation, at least for viral glycoproteins.

Our findings admit explanation by general principles and may therefore hold more generally, namely: being free from backbone hydrogen bonds increases the mutagenic potential within the receptor-binding subunit of a viral glycoprotein, and therefore deleting backbone hydrogen bonds within the constraints of molecular functionality can increase the mutagenic potential of a glycoprotein.

These considerations may be of utility for anticipating mutagenic pressure within the receptor-binding subunit of a viral glycoprotein based on a lack of backbone hydrogen bonds, and this may be of substance in general for vaccine design.

## Figures and Tables

**Table 1:** Summary statistics of variant mutagenic residues for the PDB-covered submolecule S of the SARS-CoV-2 spike glycoprotein (residues 27 to 1147) and its sub-molecules $S_{1}$ (residues 27 to 681) and $S_{2}$ (residues 682 to 1147). Averages are per residue in each molecule summed over the 15 structures in the third and fourth columns. A residue is *missing* if it is not modeled in the PDB file (interpreted as disorganized); is *absent* if it occurs in the PDB file but does not participate in either nearby backbone hydrogen bonds (along the backbone); and is *unbonded* if it is either missing or absent in at least 10 of the 15 PDB files in the database for that residue. In each case, the bar over the molecule denotes the subset of the mutated residues M of Wuhan-Hu-1 among the variants of concern or interest and those under monitoring, namely residues (5, 9, 12, 18–20, 26) 52, 67, 69–70, 75–76, 80, 95, 136, 138, 144–145, 152, 156–158, 190, 215, 243–244, 246–253, 346, 417, 449, 452, 478, 484, 490, 501, 570, 614, 641, 655, 677, 679, 681, 701, 716, 796, 859, 888, 899, 950, 982, 1027, 1071, 1092, 1101, and 1118 (1176), where the residues in parentheses are outside PDB-coverage and not reflected in this table. A residue contributes to the average free energy BFE only if it is bonded.

Mol	#Res	avg #Missing	avg #Absent	avg #Unbonded	avg BFE
S	1121	1.39	4.91	0.35	2.41
$\bar{S}$	56	4.71	5.34	0.61	2.47
S${}_{1}$	655	1.85	5.52	0.42	3.16
${\bar{S}}_{1}$	43	6.14	5.69	0.74	2.80
S${}_{2}$	466	0.73	4.05	0.25	1.60
${\bar{S}}_{2}$	13	0.00	4.15	0.15	2.14

**Table 2:** Percentage of unbonded residues for each indicated molecule. W, Δ, and *O* are the Wuhan-Hu-1, Delta, and Omicron variants, respectively, and (*) denotes the collection of variants of concern or interest, or under monitoring from [2], with mutated residues given in Table 1. It is Tulio de Oliveira’s collection of mutated residues, namely 67, 69, 70, 142–145, 211, 212, 214, 339, 371, 373, 375, 417, 440, 446, 477, 478, 484, 493, 496, 498, 501, 505, 547, 614, 655, 679, 681, 764, 796, 856, 954, 969, and 981, that serve to define *O* for our purposes here. As in Table 1, we let $\bar{S}=S\cap M$, ${\bar{S}}_{1}=S_{1}\cap M$, and ${\bar{S}}_{2}=S_{2}\cap M$ denote the collection of mutagenic residues *M* in each of the molecules S, namely $S_{1}$, and $S_{2}$, respectively, with *M* determined by (*) in the second column and by *O* in the third and fourth columns. The last column is simply the percentage of residues which are bonded in W and unbonded in Δ in each molecule.

**Mol**	W to (*)	**Δ to O**	**W to O**	**W to Δ**
S	35	40	35	30
$\bar{S}$	61	61	22	42
S${}_{1}$	42	46	42	32
${\bar{S}}_{1}$	74	67	27	43
S${}_{2}$	25	32	25	28
${\bar{S}}_{2}$	15	33	0	33

## Data Availability

See [22] for online software.

## Abbreviations

BFE	backbone free energy
BHB	backbone hydrogen bond
PDB	protein data bank
W	Wuhan-Hu-1 SARS-CoV-2 strain
β, Δ, *O*	Beta, Delta, and Omicron SARS-CoV-2 variant
